# Simultaneous detection of thalassemia, hemoglobinopathies, and G6PD variants using long-read nanopore sequencing: genetic complexity and heterogeneity in Thailand

**DOI:** 10.7717/peerj.21605

**Published:** 2026-08-11

**Authors:** Punchita Rujirachaivej, Praew Phermthong, Paweesuda Rattanakoch, Lanlana Treeravatananon, Narisara Saelee, Supasiri Chaweewattanaskul, Suthavee Boonlikit, Arisa Koyvanich, Sitthichai Panyasai, Rossarin Karnpean, Wittaya Jomoui

**Affiliations:** 1 Department of Pathology, Maha Chakri Sirindhorn Medical Center, Faculty of Medicine, Srinakharinwirot University, Ongkharak, Nakhon Nayok, Thailand; 2 Medical Genetic and Genomics Laboratory Unit, Maha Chakri Sirindhorn Medical Center, Faculty of Medicine, Srinakharinwirot University, Ongkharak, Nakhon Nayok, Thailand; 3 Faculty of Medicine, Srinakharinwirot University, Ongkharak, Nakhon Nayok, Thailand; 4 Department of Medical Technology, School of Allied Health Sciences, University of Phayao, Phayao, Thailand; 5 Research Cluster in Hematology and Genetic Diseases, Faculty of Medicine, Srinakharinwirot University, Ongkharak, Nakhon Nayok, Thailand

**Keywords:** Thalassemia, Diseases, G6PD, Long-read sequencing, Nanopore, Hemoglobinopathies

## Abstract

Thalassemia, hemoglobinopathies, and glucose-6-phosphate dehydrogenase (G6PD) deficiency represent highly prevalent and genetically heterogeneous inherited hematologic disorders or diseases in Thailand. Conventional molecular approaches require multiple targeted assays to detect deletional and non-deletional variants across globin gene clusters and the G6PD gene, resulting in increased complexity, turnaround time, and cost. We developed and analytically validated a comprehensive long-read sequencing workflow using Oxford Nanopore Technologies for simultaneous detection of pathogenic variants in *HBA 1, HBA 2, HBB*, and *G6PD*. A total of 63 genomic DNA samples with previously characterized genotypes were analyzed using multiplex long-amplicon PCR followed by nanopore sequencing and bioinformatic variant calling with Clair 3. Variant annotation was performed using Ensembl VEP, and classification followed ACMG guidelines. All samples were successfully genotyped, demonstrating 100% concordance with conventional PCR, and Sanger sequencing. The platform accurately identified large α-globin deletions, β-thalassemia mutations, structural hemoglobin variants, and major Southeast Asian G6PD variants. A total of 39 distinct variants were detected and classified. Long-read sequencing effectively resolved homologous regions between *HBA 1* and *HBA 2* and enabled accurate characterization of multilocus co-inheritance patterns. This integrated nanopore-based workflow provides a robust, high-resolution, and scalable single-platform solution for comprehensive molecular diagnosis of thalassemia, hemoglobinopathies, and G6PD deficiency in genetically heterogeneous populations.

## Introduction

Thalassemia, hemoglobinopathies, and glucose-6-phosphate dehydrogenase (G6PD) deficiency are among the most prevalent inherited hematologic disorders in Thailand and worldwide. In the Thai population, the overall thalassemia carrier rate is estimated at 30–40%. Specifically, alpha-thalassemia (α-thal) affects 20–30% of the population, beta-thalassemia (β-thal) 3–9%, and hemoglobin E (Hb E) up to 60% (Fucharoen, Winichagoon & Thonglairum, 1988; Fucharoen, 2011). G6PD deficiency shows in approximately 5–11% of individuals, predominantly affecting males due to its X-linked inheritance (Phompradit et al., 2011). Collectively, the global burden is significant, with approximately 5% of the world’s population carrying thalassemia mutations and 4.9% affected by G6PD deficiency, marking these conditions as major public health priorities (Modell & Darlison, 2008; Nkhoma et al., 2009). More recent data continue to confirm this high burden: a national thalassemia carrier screening study in Thailand (2023) reported carrier rates broadly consistent with these estimates, with regional variations reflecting population admixture patterns (Jomoui et al., 2023). Updated global epidemiological analyses have similarly reaffirmed that hemoglobin disorders constitute one of the most common monogenic disease burdens worldwide (Sadiq et al., 2024; Weatherall, 2010).

Thalassemia and hemoglobinopathies are inherited in an autosomal recessive manner and exhibit a broad clinical spectrum, ranging from asymptomatic carrier states to severe anemia and life-threatening disease (Sadiq et al., 2024). Certain thalassemia majors, including homozygous α^0^-thalassemia, homozygous β⁰-thalassemia, and compound heterozygous β⁰-thalassemia/Hb E may result in fetal anemia or neonatal death (Sadiq et al., 2024; Weatherall, 2010). In Thailand, national prevention and control programs was implemented, emphasizing carrier screening, prenatal diagnosis, and genetic counseling (Jopang et al., 2015). Molecular testing is a gold standard for detecting for α- and β-globin gene defects, including large deletions, point mutations, gene conversions, and complex structural rearrangements (Tesio & Bauer, 2023; Jaing et al., 2021; Galanello & Cao, 2011).

The G6PD gene is located on the X chromosome (Xq28). G6PD deficiency results from diverse single-nucleotide variants and small insertions or deletions within the G6PD gene. To date, more than 400 G6PD mutations have been reported worldwide, with disease severity closely linked to variant location, particularly mutations affecting amino acid residues involved in NADP^+^ binding or protein stability (Cappellini & Fiorelli, 2008; Luzzatto, Ally & Notaro, 2020). In Thailand, it has been reported in several variants, including G6PD Mahidol (c.487G>A), G6PD Viangchan (c.871G>A), G6PD Union (c.1360C>T), G6PD Canton (c.1376G>T), G6PD Kaiping (c.1388G>A), and G6PD Chinese-5 (c.1024C>T). This extensive genetic heterogeneity has driven the development and adoption of various laboratory diagnostic methods, ranging from targeted molecular assays to sequencing-based approaches, which form the foundation of current screening and diagnostic strategies (Cappellini & Fiorelli, 2008).

Despite well-established screening programs, molecular diagnosis of thalassemia and hemoglobinopathies remains challenging due to extensive genetic heterogeneity. α-thalassemia is frequently caused by large gene deletions, whereas β-thalassemia arises from a wide spectrum of point mutations, small insertions, or deletions. Hemoglobin variants, including Hb E, Hb Constant Spring, and other unstable hemoglobin, further increase diagnostic complexity. Conventional molecular techniques, such as gap-PCR, allele-specific PCR, reverse dot blot hybridization, and high-resolution melting analysis, are widely used for detecting common mutations in Thailand. However, these targeted approaches are limited to predefined variants and may fail to detect rare or novel variants. Consequently, multiple assays are often required, increasing turnaround time, labor intensity, and overall cost (Jomoui et al., 2023; Yamsri et al., 2011; Laosombat et al., 1992; Prajantasen, Fucharoen & Fucharoen, 2015; Charoenkwan et al., 2017; Sutcharitchan et al., 1995). Likewise, molecular characterization of G6PD deficiency exhibits diagnostic constraints. Although targeted genotyping panels can identify common regional variants, rare or previously unreported variants may therefore remain undetected (Cappellini & Fiorelli, 2008; Luzzatto, Ally & Notaro, 2020).

Advances in next-generation sequencing (NGS) technologies have improved variant detection; however, short-read sequencing platforms may still challenges in resolving repetitive regions, GC-rich sequences, and homologous gene clusters. The high sequence similarity between *HBA1* and *HBA2* genes, as well as the presence of pseudogenes and recombination events, further complicates accurate variant calling and haplotype phasing using short-read data. Recently, long-read sequencing, particularly Oxford Nanopore Technologies (ONT), provides an opportunity to overcome these limitations (Logsdon, Vollger & Eichler, 2020; Zheng et al., 2023). Given the high prevalence and marked genetic heterogeneity of thalassemia, hemoglobin variants, and G6PD deficiency in Thailand, a comprehensive, single-platform molecular diagnostic approach is urgently needed. Therefore, the present study aimed to develop, optimize, and analytically evaluate a long-read nanopore sequencing workflow for simultaneous detection of pathogenic variants in the globin gene clusters and the G6PD gene. We further assessed its performance compared with conventional molecular methods and explored its potential application in routine diagnostic practice within a genetically heterogeneous Thai population.

## Materials and Methods

### Samples

**Figure 1:**
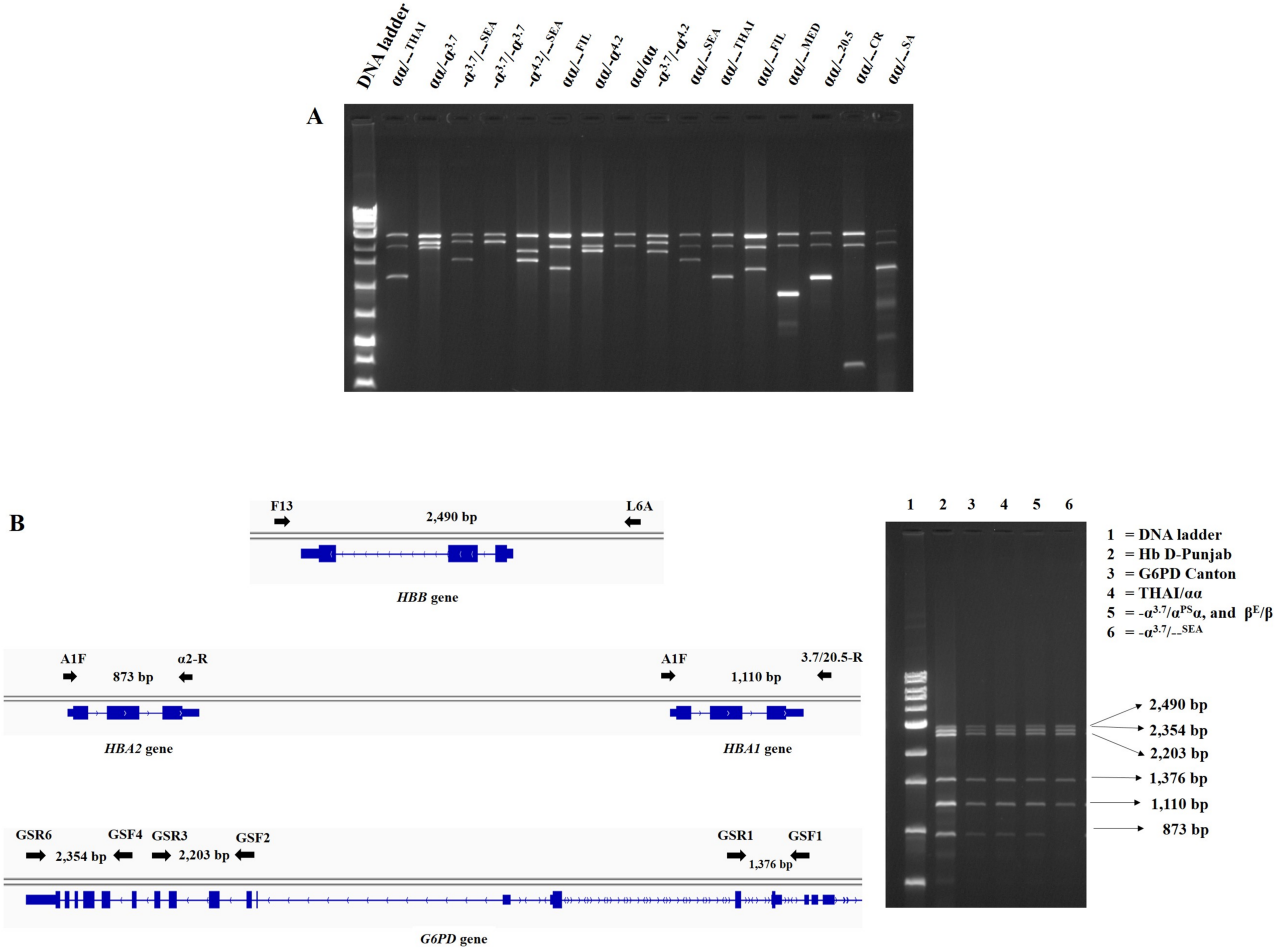
Multiplex PCR strategy for simultaneous detection of hemoglobinopathies and G6PD variants. (A) Representative agarose gel electrophoresis demonstrating amplification profiles of deletional α^0^-thalassemia including SEA, THAI, MED, Chiang Rai, and South Africa deletions, 20.5 kb deletion and α^+^-thalassemia including 3.7 and 4.2 kb deletions using gap-PCR in conventional. (B) Schematic representation of primer positions and expected amplicon sizes for *HBB* (2,490 bp), *HBA 2* (873 bp), *HBA 1* (1,110 bp), and *G6PD* (2,354, 2,203, and 1,376 bp) genes for multiplex long-amplification PCR and the validation gel showing multiplex PCR products are indicated on the right.

Ethical approval of the study was obtained from the Institutional Review Board of Srinakharinwirot University (SWU), Thailand (SWUEC-683033) based on the Declaration of Helsinki, Belmont Report, International Conference on Harmonization in Good Clinical Practice (ICH-GCP), International Guidelines for Human Research, along with laws and regulations of Thailand. In this study, we use leftover specimens that are informed by a broad consent form with the first collected specimen. All participants provided written informed consent at the time of initial sampling. A total of 63 leftover genomic DNA samples was recruited and genotyped with routine analysis. Inclusion criteria were: (1) residual leftover genomic DNA samples from patients referred for routine testing; (2) samples with previously confirmed genotypes by at least one validated conventional molecular method (gap-PCR, allele-specific PCR, or Sanger sequencing); and (3) sufficient DNA quantity (≥20 ng/µL) for multiplex PCR amplification. Exclusion criteria was samples with degraded DNA as assessed by agarose gel electrophoresis. Identifications of deletional α^0^-thalassemia including SEA, THAI, MED, Chiang Rai, and South Africa deletions, 20.5 kb deletion and α^+^-thalassemia including 3.7 and 4.2 kb deletions, were routinely performed using gap-PCR (Fig. 1A). Whereas Hb Constant Spring (CS) and Hb Paksé (PS) as non-deletional α^+^-thalassemia were performed based on allele-specific PCR methods as described elsewhere (Jomoui et al., 2023, 2020; Srivorakun et al., 2014). Identification of β-thalassemia mutations in Thailand was performed with allele-specific PCR or direct DNA sequencing on an ABI PRISM™ 3,130 XLanalyzer (Applied Biosystems, Foster City, CA, USA) as previously described (Panichchob et al., 2021; Sirichotiyakul, Saetung & Sanguansermsri, 2003). G6PD mutations were identified by direct DNA sequencing of the coding region as previously described (Dallol et al., 2012).

### Multiplex long-amplification PCR

Simultaneous multiplex long-amplification PCR for covering *HBA1, HBA2, HBB, and G6PD* genes was designed and developed using primers (Jomoui et al., 2023; Dallol et al., 2012; Tepakhan & Jomoui, 2021) that have been reported (Fig. 1B). The 50 uL of PCR reaction contained 0.2 mM of dNTPs, 1.5 mM of MgCl_2_, 1M betain, 0.1 × DTT, 0.125 uM of 3.7/20.5R primer, 0.15 uM of each A17F and α-2R primers, 0.48 uM of each GSF1, and GSR1 primers, 0.24 uM of each GSF2, and GSR3 primers, 0.54 uM of each GSF4, and GSR6 primers, 2.4 uM of L6A primer, 2.2 uM of F13 primer, 3 unit Gotaq Flexi DNA polymerase with 1 × PCR buffer (Promega), and 30–70 ng gDNA. Thermal cycling was performed on PCR machine (Sensoquest), starting with hold stage (97 °C for 5 min) for activating the Taq DNA polymerase, followed by 26 cycles of PCR (97 °C for 1 min, 62 °C for 3 min, 72 °C for 2 min). The final extension is 72 °C for 2 min. The visualization of the PCR amplification product was performed on 1% agarose gel electrophoresis with gel documentation systems (gel docs) as shown in Fig. 1B.

### Library preparation, sequencing, and base calling based on ONT

ONT-library preparation was performed using ligation sequencing amplicons–Native Barcoding Kit V14 (SQKNBD114, Oxford Nanopore Technologies, Oxford, UK), following the manufacturer’s protocol. Briefly, amplified DNA underwent end-repair and dA-tailing using the NEBNext Ultra II End Repair/dA-Tailing Module (New England Biolabs, Ipswich, MA, USA). After purification using AMPure XP beads (Beckman Coulter, Brea, CA, USA), native barcodes were ligated to each sample using Blunt/TA Ligase master mix (New England Biolabs, Ipswich, MA, USA). Barcoded samples were pooled equimolarly and subjected to adapter ligation using ONT Adapter Mix and Quick T4 DNA Ligase. The final library was purified using AMPure XP beads, quantified, and loaded onto FLOW CELL (R10.4.1) mounted on a MinION Mk1C device (Oxford Nanopore Technologies, Oxford, UK). Sequencing was performed according to the manufacturer’s standard operating procedure. The run configuration was set using the MinKNOW software interface. Real-time basecalling was enabled using the high-accuracy model (Guppy v4.3.0, 400 bps configuration). Barcode demultiplexing was performed during the run with barcode splitting enabled, and all barcodes were selected for analysis. Sequencing runs were configured with a maximum duration of 12 h or until pore exhaustion. The data output formats included FASTQ, BAM, and raw signal data in POD5. FASTQ compression was enabled to optimize storage capacity. Quality filtering was applied with a minimum Q score threshold of 9. All sequences (FASTQ) were deposited in a public database BioProject (PRJNA1439314) with sequence read archive (SRA) on NCBI database.

### Bioinformatics analysis

Variant calling was performed using Clair3 (version current at time of analysis), a deep learning–based variant caller optimized for long-read sequencing data. Clair3 was executed within a Docker container environment (hkubal/Clair3:latest) to ensure computational reproducibility and consistent dependency management. Analysis was restricted to target regions corresponding to *HBB, HBA1, HBA2*, and *G6PD* genes using a BED file (target_genes.bed) specifying genomic coordinates of interest. This targeted approach reduced computational burden and minimized off-target variant calls. For each sample, BAM files were processed individually using the following parameters: reference genome: GRCh38 (hg38.fa). Clair3 was executed using the run_clair3.sh pipeline with default settings optimized for ONT data. Variant calling included detection of single-nucleotide variants (SNVs) and small insertions/deletions (indels). Output files were generated in VCF format for each sample. The computational workflow was automated using a Bash script to iterate over all BAM files within the designated input directory. Output directories were generated per sample to ensure traceability and structured data management. All analyses were performed in a Linux-based environment (WSL2) with Docker support enabled.

Variant annotation was performed using Ensembl Variant Effect Predictor (VEP, Ensembl release; https://www.ensembl.org), based on the GRCh38 (hg38) reference genome. The VEP tool was used to determine the functional consequences of identified variants at the transcript and protein levels, including classification as synonymous, missense, nonsense, splice-site, frameshift, or in-frame insertion/deletion variants. Aligned BAM files and corresponding VCF outputs were visually inspected using Integrative Genomics Viewer 2.19.7 (IGV, Broad Institute, Cambridge, MA, USA) to confirm variant calls. Furthermore, all annotated variants were interpreted and classified according to the standards and guidelines of the American College of Medical Genetics and Genomics (ACMG). Variant classification was performed using the Franklin by Genoox platform (Franklin, Genoox Ltd., Tel Aviv, Israel), which integrates curated databases, population frequency data, computational prediction tools, and published literature to support evidence-based variant interpretation. Each variant was evaluated under the five-tier ACMG classification system: pathogenic (P), likely pathogenic (LP), variant of uncertain significance (VUS), likely benign (LB), and benign (B).

## Results

### Study cohort and genotypic spectrum

A total of 63 genomic DNA samples with previously characterized thalassemia, hemoglobinopathy, and G6PD genotypes by conventional molecular methods were successfully analyzed using the ONT-based long-read sequencing workflow. All samples yielded adequate amplicon PCR products covering *HBA1, HBA2, HBB*, and *G6PD*, as demonstrated by clear and specific amplicon bands on agarose gel electrophoresis (Fig. 1B). No amplification failure was observed across the cohort.

The genotypic distribution reflected the marked genetic heterogeneity typical of the Thai population. Among the 63 samples, α-globin genotypes comprised normal (αα/αα), single α^+^-thalassemia deletions (-α^3.7^, -α^4.2^), compound heterozygous deletional states (*e.g*., -α^3.7^/-α^4.2^), α^0^-thalassemia deletions ( ${\textrm{--}}$^SEA^, ${\textrm{--}}$^THAI^, ${\textrm{--}}$^FIL^), and Hb variants including Hb Constant Spring (CS), Hb Pakse (PS), Hb Hekinan, Hb Q-Thailand, Hb Westmead, Hb Grey Lynn, Hb J-Singapore, Hb G-Georgia, and others (Table 1).

**Table 1:** A total of 63 samples with differences genotypes of thalassemia and G6PD identified by ONT.

Thalassemia and G6PD genotypes			ONT-based long read sequencing (NGS)		
*HBA1/HBA2*	*HBB*	*G6PD*	*HBA1/HBA2*	*HBB*	*G6PD*
αα/αα	β^17^/β	Mahidol	–	HBB:c.52A>T	G6PD:c.487G>A
αα/αα	β^19^/β	Wild type	–	HBB:c.59A>G	–
αα/αα	β^-31^/β	Wild type	–	HBB:c.-81A>G	–
αα/αα	β^41/42^/β	Wild type	–	HBB:c.126_129del	–
αα/αα	β^-50^/β	Wild type	–	HBB:c.-100G>A	–
αα/αα	β^-50^/β^17^	Canton	–	HBB:c.−100G>A HBB:c.52A>T	G6PD:c.1376G>T
αα/αα	β^71/72^/β	Viangchan	–	HBB:c.217dup	G6PD:c.871G>A
αα/αα	β^71/72^/β^E^	Wild type	–	HBB:c.217dup HBB:c.79G>A	–
αα/αα	β^8/9^/β	Wild type	–	HBB:c.27dup	–
αα/αα	β^IVS1-1^/β^New York^	Wild type	–	HBB:c.92+1G>T HBB:c.341T>A	–
αα/αα	β^IVS1-5^/β^E^	Wild type	–	HBB:c.92+5G>C HBB:c.79G>A	–
αα/αα	β^IVS1-5^/β	Wild type	–	HBB:c.92+5G>C	–
αα/αα	β^IVS2-654^/β	Wild type	–	HBB:c.316-197C>T	–
αα/αα	β/β	Canton	–	–	G6PD:c.1376G>T
αα/αα	β^E^/β	Viangchan	–	HBB:c.79G>A	G6PD:c.871G>A
αα/αα	β^E^/β^E^	Viangchan	–	HBB:c.79G>A	G6PD:c.871G>A
αα/αα	β^D-Punjab^/β	Wild type	–	HBB:c.364G>C	–
αα/αα	β^E^/β	Wild type	–	HBB:c.79G>A	–
αα/αα	β^E^/β^Tak^	Wild type	–	HBB:c.79G>A HBB:c.440_441dup	–
αα/αα	β^J-Bangkok^/β	Wild type	–	HBB:c.170G>A	–
αα/αα	β^Korle Bu^/β	Wild type	–	HBB:c.220G>A	–
αα/αα	β^Tak^/β	Wild type	–	HBB:c.440_441dup	–
αα/αα	β^S^ β	Wild type	–	HBB:c.20A>T	–
-α^3.7^/αα	β^-28^/β^E^	Wild type	–	HBB:c.-78A>G HBB:c.79G>A	–
-α^3.7^/αα	β^35^/β	Wild type	–	HBB:c.108C>A	–
-α^3.7^/αα	β^41/42^/β	Wild type	–	HBB:c.126_129del	–
-α^3.7^/αα	β/β	Union	–	–	G6PD:c.1360C>T
-α^4.2^/αα	β^E^/β	Viangchan	–	HBB:c.79G>A	G6PD:c.871G>A
-α^4.2^/αα	β^D-Punjab^/β	Wild type	–	HBB:c.364G>C	–
-α^3.7^/-α ^3.7^	β^IVS1-1^/β	Wild type	–	HBB:c.92+1G>T	–
-α^3.7^/-α ^3.7^	β^E^/β^E^	Wild type	–	HBB:c.79G>A	–
-α ^3.7^/-α ^4.2^	β^E^/β	Wild type	–	HBB:c.79G>A	–
${\textrm{--}}$^SEA^/αα	β^Raleigh^/β	Wild type	–	HBB:c.5T>C	–
${\textrm{--}}$^SEA^/αα	β^New York^/β	Wild type	–	HBB:c.341T>A	–
${\textrm{--}}$^SEA^/αα	β/β	Wild type	–	–	–
${\textrm{--}}$^THAI^/αα	β/β	Wild type	–	–	–
${\textrm{--}}$^FIL^/αα	β/β	Wild type	–	–	–
-α^3.7^/ ${\textrm{--}}$^SEA^	β/β	Mahidol	–	–	G6PD:c.487G>A
-α^3.7^/ ${\textrm{--}}$^SEA^	β^C^/β	Wild type	–	HBB:c.19G>A	–
-α^4.2^/ ${\textrm{--}}$^SEA^	β/β	Wild type	–	–	–
α^CS^α/αα	β^E^/β	Wild type	HBA2:c.427T>C	HBB:c.79G>A	–
α^PS^α/αα	β^E^/β	Wild type	HBA2:c.429A>T	HBB:c.79G>A	–
αα^Grey Lynn^/αα	β/β	Wild type	HBA1:c.274C>T	–	–
αα^Hekinan^/αα	β/β	Viangchan	HBA1:c.84G>T	–	G6PD:c.871G>A
α^Jax^α/αα	β/β	Wild type	HBA2:c.44G>C	–	–
αα^J-Buda^/αα	β/β	Wild type	HBA1:c.186G>T	–	–
α^J-Sigapore^α/αα	β/β	Wild type	HBA2:c.239C>G	–	–
α^Nakhon Ratchasima^α/αα	β^41/42^/β	Viangchan	HBA2:c.191C>T	HBB:c.126_129del	G6PD:c.871G>A
α^CS^α/α^CS^α	β^C^/β	Viangchan	HBA2:c.427T>C	HBB:c.19G>A	G6PD:c.871G>A
α^CS^α/αα^Hekinan^	β^E^/β	Wild type	HBA1:c.84G>T	HBB:c.79G>A	–
α^CS^α/αα^Lansing ramathibodi^	β/β	Wild type	HBA1:c.264C>G	–	–
-α^3.7^/α^CS^α	β^Pyrgos^/β	Wild type	HBA2:c.427T>C	–	–
-α^3.7^/α^CS^α	β/β	Wild type	HBA2:c.427T>C	–	–
-α^3.7^/α^PS^α	β^E^/β	Wild type	HBA2:c.429A>T	HBB:c.79G>A	–
-α^Doi-Saket(3.7)^/αα	β/β	Wild type	HBA1:c.30C>A	–	–
-α^Q-Thailand (4.2)^/α^CS^α	β/β	Wild type	HBA1:c.223G>C HBA2:c.427T>C	–	–
-α^Q-Thailand (4.2)^/αα	β/β	Wild type	HBA1:c.223G>C	–	–
${\textrm{--}}$^SEA^/α^CS^α	β^J-Bangkok^/β	Wild type	HBA2:c.427T>C	HBB:c.170G>A	–
${\textrm{--}}$^SEA^/α^CS^α	β/β	Wild type	HBA2:c.427T>C	–	–
${\textrm{--}}$^SEA^/α^G-Georgia^α	β/β	Wild type	HBA2:c.287C>T	–	–
${\textrm{--}}$^SEA^/αα^Lansing ramathibodi^	β^E^/β	Canton	HBA1:c.264C>G	HBB:c.79G>A	G6PD:c.1376G>T
${\textrm{--}}$^SEA^/α^Westmead^α	β/β	Viangchan	HBA2:c.369C>G	–	G6PD:c.871G>A
${\textrm{--}}$^SEA^/α^Westmead^α	β^E^/β	Wild type	HBA2:c.369C>G	HBB:c.79G>A	–

For β-globin, we identified a wide spectrum of β-thalassemia mutations and structural Hb variants. These included common Southeast Asian β-thalassemia mutations such as β^0^ and β^+^ alleles (*e.g*., β^17^ [c.52A>T], β^19^ [c.59A>G], β^41/42^ [c.126_129del], β^IVS1-1^ [c.92+1G>T], β^IVS1-5^ [c.92+5G>C], β^IVS2-654^ [c.316–197C>T], β^71/72^ [c.217dup], β^35^ [c.108C>A], β^-28^ [c.-78A>G], β^-31^ [c.-81A>G], and β^-50^ [c.-100G>A]), as well as structural variants including Hb E (c.79G>A), Hb D-Punjab (c.364G>C), Hb Tak (c.440_441dup), Hb J-Bangkok (c.170G>A), Hb Korle Bu (c.220G>A), Hb C (c.19G>A), Hb S (c.20A>T), Hb New York (c.341T>A), and Hb Raleigh (c.5T>C) (Table 1). Both heterozygous and compound heterozygous combinations were detected, including clinically significant genotypes such as β^E^/β^E^, β^E^/β^0^, and β-thalassemia with co-inherited α-thalassemia.

Whereas G6PD variants identified included the predominant Southeast Asian variants Mahidol (c.487G>A), Viangchan (c.871G>A), Canton (c.1376G>T), and Union (c.1360C>T), observed either as isolated findings or co-inherited with thalassemia genotypes.

### Analytical performance of ONT long-read sequencing

**Figure 2:**
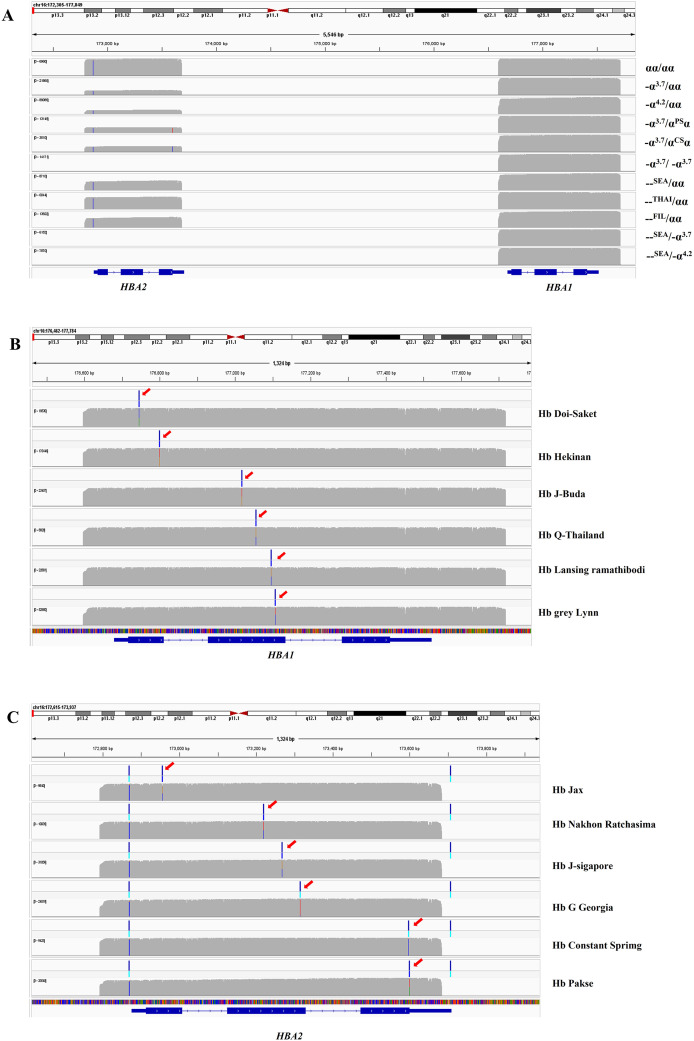
Long-read nanopore sequencing analysis of α-globin structural variants and non-deletional mutations. (A) Integrative Genomics Viewer (IGV) snapshots demonstrating read-depth patterns across the α-globin gene cluster (*HBA2* and *HBA1*) for representative genotypes. Deletions are visualized as reduced read coverage spanning breakpoint regions. IGV views of variants in *HBA1* (B) and in *HBA2* (C). Variant loci are marked by red arrows. Gene structures are shown at the bottom of each panel.

**Figure 3:**
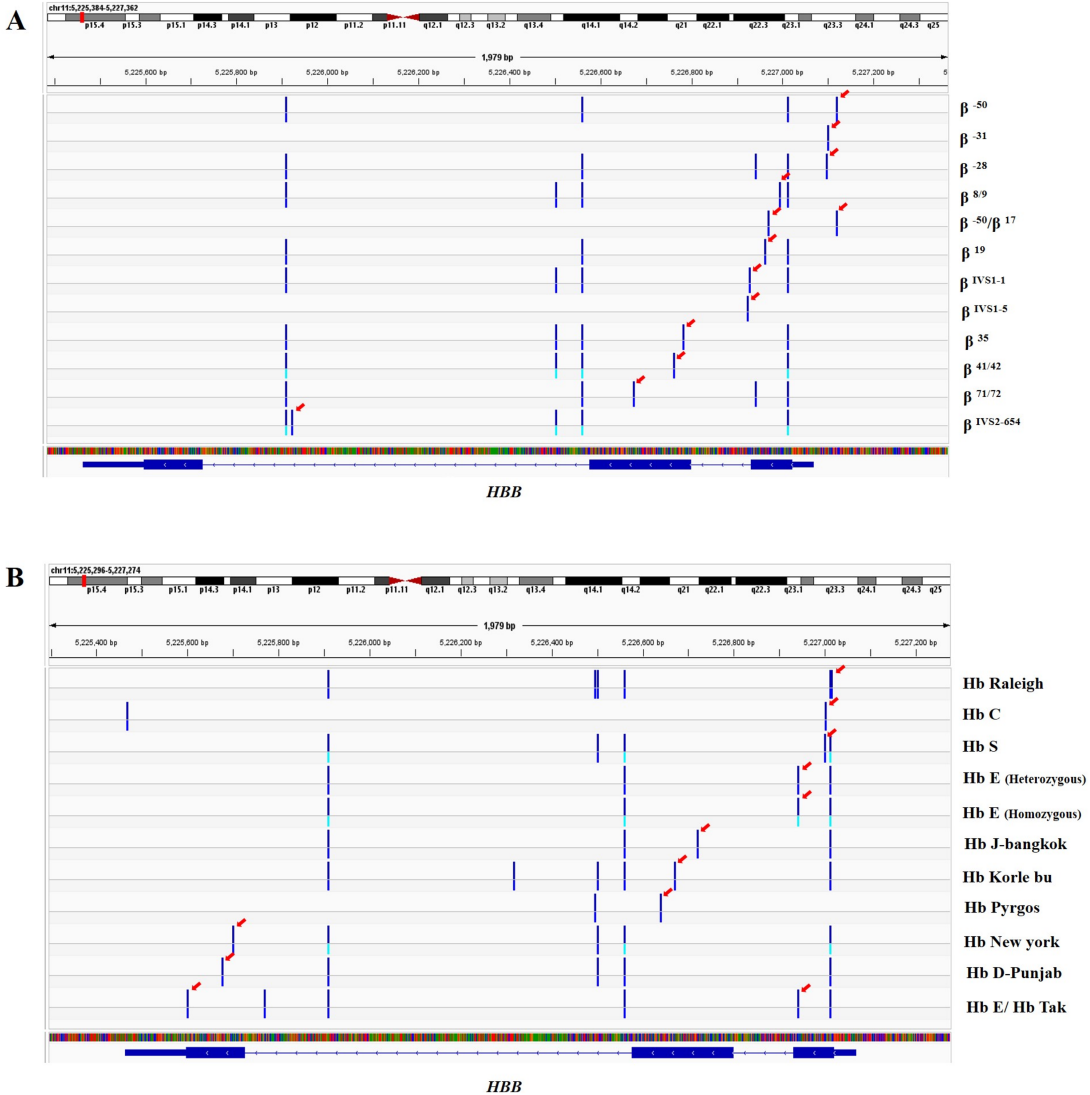
Long-read nanopore sequencing analysis of β-globin (*HBB*) variants. (A) Integrative Genomics Viewer (IGV) snapshots showing representative β-thalassemia mutations across the *HBB* gene. Variant positions are indicated by red arrows. (B) IGV views of structural hemoglobin variants in *HBB*. Red arrows indicate nucleotide substitutions. Gene structures are shown at the bottom of each panel.

**Figure 4:**
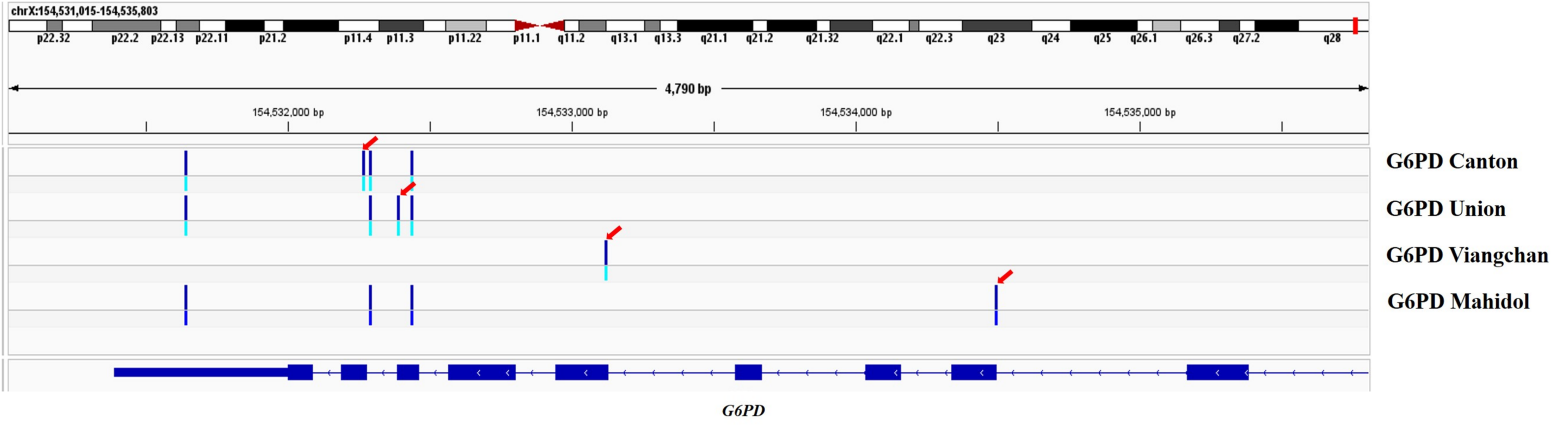
Long-read nanopore sequencing analysis of common G6PD variants. Integrative Genomics Viewer (IGV) snapshots showing representative pathogenic variants in the G6PD gene (chrX), including G6PD Canton, G6PD Union, G6PD Viangchan, and G6PD Mahidol. Red arrows indicate the nucleotide substitution sites.

All previously characterized variants identified by conventional gap-PCR, allele-specific PCR, or Sanger sequencing were concordantly detected by the ONT workflow. This included large α-globin deletions (*e.g*., ${\textrm{--}}$^SEA^, ${\textrm{--}}$^THAI^, ${\textrm{--}}$^FIL^, -α^3.7^, -α^4.2^), Small insertions/deletions in *HBB* (*e.g*., c.126_129del, and c.217dup, c.440_441dup). Promoter and splice-site variants (*e.g*., c.-78A>G, c.-81A>G, c.92+1G>T, c.92+5G>C, c.316-197C>T) and Missense variants in *HBA1, HBA2, HBB*, and *G6PD*. Variant visualization using IGV confirmed high-confidence base-calling and clear alignment to the GRCh38 reference genome (Figs. 2–4). The high sequence homology between *HBA1* and *HBA2* did not result in misalignment or misclassification, and gene-specific variants were accurately resolved.

Deletional α-thalassemia genotypes demonstrated characteristic read-depth reduction and breakpoint-spanning reads consistent with expected structural configurations (Fig. 2A). For β-thalassemia, both SNVs and indels were clearly identified with distinct allele fractions consistent with heterozygous or compound heterozygous states (Fig. 3). G6PD variants were similarly visualized with unambiguous variant peaks and balanced allele representation in heterozygous female samples (Fig. 4).

### Variant annotation and ACMG classification

A total of 39 distinct variants (SNVs and indels) across *HBA1, HBA2, HBB*, and *G6PD* were identified and annotated using Ensembl VEP and classified according to ACMG guidelines *via* the Franklin platform (Table 2). Among α-globin variants, Hb Constant Spring (*HBA2*:c.427T>C) was classified as pathogenic, and Hb Pakse (*HBA2*:c.429A>T) as likely pathogenic. Several structural Hb variants such as Hb Lansing Ramathibodi (*HBA1*:c.264C>G) were classified as likely pathogenic, whereas others (*e.g*., Hb Hekinan, Hb Q-Thailand, Hb Grey Lynn, Hb Westmead, and Hb G-Georgia) were categorized as variants of uncertain significance (VUS) under ACMG criteria. For β-globin, multiple classical β-thalassemia mutations were classified as pathogenic, including β^17^, β^19^, β^8/9^, β^41/42^, β^71/72^, β^IVS1-1^, β^IVS1-5^, β^IVS2-654^, β^-28^, and Hb S. Whereas Hb E (c.79G>A) was classified as likely pathogenic in accordance with its well-established disease association. Several structural variants (*e.g*., Hb J-Bangkok, Hb Korle Bu, Hb Pyrgos, Hb Raleigh) were categorized as VUS due to limited clinical evidence despite documented molecular changes. All detected G6PD variants were classified as likely pathogenic (Mahidol, Viangchan, and Union) or pathogenic (Canton), consistent with previously reported functional impact.

**Table 2:** Single Nucleotide Variants (SNVs) and insertions/deletions (indels or dels) of *HBA1, HBA2, HBB*, and *G6PD* genes were evaluated and classified by ONT and ACMG (*n* = 39 variants).

Variants	HGVS	SNP ID	Franklin ACMG classification
Hb Doi-Saket	HBA1:c.30C>A	–	Variant of uncertain significance
Hb Hekinan	HBA1:c.84G>T	rs41530750	Variant of uncertain significance
Hb J-Buda	HBA1:c.186G>T	rs281864775	Variant of uncertain significance
Hb Q-Thailand	HBA1:c.223G>C	rs28928875	Variant of uncertain significance
Hb Lansing ramathibodi	HBA1:c.264C>G	rs1318210119	Likely Pathogenic
Hb grey Lynn	HBA1:c.274C>T	rs17407508	Variant of uncertain significance
Hb Jax	HBA2:c.44G>C	–	Variant of uncertain significance
Hb Nakhon Ratchasima	HBA2:c.191C>T	rs281864848	Variant of uncertain significance
Hb J-sigapore	HBA2:c.239C>G	rs281860603	Variant of uncertain significance
Hb G Georgia	HBA2:c.287C>T	rs281864880	Variant of uncertain significance
Hb Westmead	HBA2:c.369C>G	rs41479347	Variant of uncertain significance
Hb Constant Spring	HBA2:c.427T>C	rs41464951	Pathogenic
Hb Pakse	HBA2:c.429A>T	rs41412046	Likely Pathogenic
β^-50^	HBB:c.-100G>A	rs281864524	Variant of uncertain significance
β^-31^	HBB:c.-81A>G	rs33981098	Pathogenic
β^-28^	HBB:c.-78A>G	rs33931746	Pathogenic
Hb Raleigh	HBB:c.5T>C	rs33949930	Variant of uncertain significance
Hb C	HBB:c.19G>A	rs33930165	Likely Pathogenic
Hb S	HBB:c.20A>T	rs334	Pathogenic
β^8/9^	HBB:c.27dup	rs35699606	Pathogenic
β^17^	HBB:c.52A>T	rs33986703	Pathogenic
β^19^	HBB:c.59A>G	rs33972047	Pathogenic
Hb E	HBB:c.79G>A	rs33950507	Likely Pathogenic
β^IVS1-1^	HBB:c.92+1G>T	rs33971440	Pathogenic
β^IVS1-5^	HBB:c.92+5G>C	rs33915217	Pathogenic
β^35^	HBB:c.108C>A	rs33982568	Pathogenic
β^41/42^	HBB:c.126_129del	rs80356821	Pathogenic
Hb J-Bangkok	HBB:c.170G>A	rs34439278	Variant of uncertain significance
β^71/72^	HBB:c.217dup	rs33969853	Pathogenic
Hb Korle Bu	HBB:c.220G>A	rs33945705	Variant of uncertain significance
Hb Pyrgos	HBB:c.251G>A	rs1803195	Variant of uncertain significance
β^IVS2-654^	HBB:c.316-197C>T	rs34451549	Pathogenic
Hb New York	HBB:c.341T>A	rs34484056	Likely Pathogenic
Hb D-Punjab	HBB:c.364G>C	rs33946267	Pathogenic
Hb Tak	HBB:c.440_441dup	rs33999427	Pathogenic
G6PD Union	G6PD:c.1360C>T	rs398123546	Likely Pathogenic
G6PD Canton	G6PD:c.1376G>T	rs72554665	Pathogenic
G6PD Mahidol	G6PD:c.487G>A	rs137852314	Likely Pathogenic
G6PD Viangchan	G6PD:c.871G>A	rs137852327	Likely Pathogenic

### Co-inheritance patterns and genetic complexity

The long-read approach enabled simultaneous identification of complex genotypes involving co-inheritance of α-thalassemia, β-thalassemia, hemoglobin variants, and G6PD deficiency within a single assay. Representative combinations included ${\textrm{--}}$^SEA^/α^CS^α with β^J-Bangkok^/β, α^CS^α/α^CS^α with β^C^/β and G6PD Viangchan, -α^3.7^/ ${\textrm{--}}$^SEA^ with G6PD Mahidol, α^Nakhon Ratchasima^α/αα with β^41/42^/β and G6PD Viangchan, and β^E^/β^E^ with G6PD Viangchan. These findings underscore the substantial allelic heterogeneity and multilocus inheritance patterns present in the Thai population. Importantly, all compound and co-inherited genotypes were accurately resolved without the need for multiple sequential assays.

## Discussion

In this study, we developed and analytically validated a comprehensive ONT-based long-read sequencing workflow for simultaneous detection of pathogenic variants in *HBA1, HBA2, HBB*, and *G6PD* genes in a genetically heterogeneous Thai cohort. All 63 samples were successfully genotyped with complete concordance (100%) with prior conventional molecular methods, including gap-PCR, allele-specific PCR, and Sanger sequencing. Thailand and Southeast Asia represent one of the most genetically complex regions for hemoglobin disorders worldwide, with high carrier frequencies of α-thalassemia, β-thalassemia, and Hb E, as well as substantial prevalence of G6PD deficiency (Fucharoen, Winichagoon & Thonglairum, 1988; Fucharoen, 2011; Phompradit et al., 2011). The broad spectrum of variants observed in our cohort including ${\textrm{--}}$^SEA^, ${\textrm{--}}$^THAI^, -α^3.7^, -α^4.2^, Hb Constant Spring, Hb Pakse, multiple β-thalassemia alleles (β^17^, β^41/42^, β^IVS1-1^, β^IVS2-654^), Hb E, Hb D-Punjab, Hb Tak, and four major G6PD variants (Mahidol, Viangchan, Canton, and Union) which closely reflects previously reported molecular epidemiology studies in Thailand (Phompradit et al., 2011; Jomoui et al., 2023, 2020; Panichchob et al., 2021).

However, the current assay is designed to detect point mutations, small indels, and common alpha-globin deletions, but does not cover large beta-globin cluster deletions. We propose that integration of multiplex ligation-dependent probe amplification (MLPA) targeting the beta-globin cluster or in future iterations, a capture-based long-read sequencing approach spanning the full ~70 kb beta-globin locus, would be necessary for comprehensive hemoglobinopathy screening. Furthermore, the current workflow reliably identifies common alpha-thalassemia deletions *via* read-depth reduction and breakpoint-spanning reads; however, rare or novel deletions require additional confirmatory methods such as MLPA or targeted breakpoint sequencing for precise characterization. Notably, the HS-40 regulatory element (~40 kb upstream of the alpha-globin cluster) is not covered by the present amplicon design, meaning deletions that selectively remove HS-40 without disrupting structural genes would be missed despite causing severe alpha-thalassemia. Future iterations should incorporate HS-40 coverage through broader tiling amplicons or a capture-based approach.

A major challenge in hemoglobinopathy diagnostics is the necessity to use multiple complementary assays. Deletional α-thalassemia typically requires gap-PCR or MLPA (Jomoui et al., 2023), whereas β-thalassemia and G6PD variants are often assessed by targeted PCR panels or Sanger sequencing (Panichchob et al., 2021; Sirichotiyakul, Saetung & Sanguansermsri, 2003; Dallol et al., 2012). Such stepwise strategies increase cost, turnaround time, and the risk of incomplete diagnosis. In contrast, our ONT-based approach demonstrated robust detection of large structural deletions (*e.g*., ${\textrm{--}}$^SEA^, -α^3.7^), confirmed by breakpoint-spanning reads and characteristic read-depth changes, promoter and splice-site variants, frame-shift, duplication variants, and missense hemoglobin variants and G6PD mutations. These findings are consistent with prior reports demonstrating the superiority of long-read sequencing in resolving structural variants and homologous genomic regions (Logsdon, Vollger & Eichler, 2020; Zheng et al., 2023). The high sequence homology between *HBA1* and *HBA2* is a known limitation of short-read platforms, which did not result in misalignment or variant misclassification in the study.

Short-read NGS has improved variant detection in inherited hematologic disorders but remains limited in resolving repetitive regions, homologous genes, and structural rearrangements (Logsdon, Vollger & Eichler, 2020). In contrast, ONT long-read sequencing offers direct phasing information, improved structural variant detection, and simplified workflow integration. Our study extends these observations to common hemoglobinopathies and demonstrates that even in a targeted amplicon-based design, long-read sequencing can achieve full concordance with gold-standard methods while reducing assay fragmentation. Importantly, unlike whole-genome long-read approaches, which may remain cost-prohibitive for routine screening, our multiplex long-amplicon design represents a cost-conscious, scalable solution adaptable to regional diagnostic laboratories.

We identified 39 distinct variants and classified them according to ACMG criteria. Classical β-thalassemia mutations and Hb S were categorized as pathogenic, consistent with well-established disease mechanisms (Tesio & Bauer, 2023; Jaing et al., 2021). Specifically, classical β-thalassemia mutations disrupt *HBB* gene expression through distinct molecular pathways: nonsense mutations (*e.g*., β^17^: c.52A>T) introduce premature stop codons leading to nonsense-mediated mRNA decay and absent β-globin chain synthesis (β^0^ phenotype); frameshift mutations (*e.g*., β^41/42^: c.126_129del, β^71/72^: c.217dup) alter the reading frame, producing truncated or rapidly degraded protein; splice-site mutations (*e.g*., β^IVS1-1^, β^IVS2-654^) impair normal pre-mRNA processing, resulting in absent or markedly reduced functional mRNA. Hb S (c.20A>T; Glu6Val substitution) causes erythrocyte sickling under hypoxic conditions due to polymerization of deoxygenated HbS tetramers, underpinning the vaso-occlusive and hemolytic pathophysiology of sickle cell disease (Tesio & Bauer, 2023; Jaing et al., 2021). These mechanistic features directly correspond to the genotypes identified in the present cohort and justify their pathogenic classification under ACMG criteria. Hb E was classified as likely pathogenic, aligning with its dual role as a structural variant and mild β^+^-thalassemia allele (Fucharoen, 2011; Yamsri et al., 2011). Interestingly, several structural hemoglobin variants (*e.g*., Hb J-Bangkok, Hb Korle Bu, Hb Raleigh) were categorized as variants of uncertain significance (VUS). This highlights an important issue in hemoglobinopathy genomics; although many variants are historically described in the literature, formal ACMG classification may still be limited by insufficient functional or segregation data. The VUS designations in this study reflect multiple converging limitations: absence of formal *in vitro* functional data for rare structural variants (*e.g*., Hb J-Bangkok, Hb Korle Bu, Hb Pyrgos, Hb Raleigh) precluding application of ACMG criteria PS3/BS3; sparse allele frequency data in global databases (gnomAD) for Southeast Asian-specific variants, limiting use of PM2/BA1 criteria; lack of published family co-segregation studies supporting PP1; and variable genotype–phenotype correlations, as some variants are clinically silent in heterozygotes but potentially significant in compound heterozygous states. Collectively, VUS classification does not imply benign status, and future functional characterization and longitudinal follow-up are warranted for reclassification, particularly for variants prevalent in the Thai population. Similar observations have been noted in previous large-scale variant databases of hemoglobin variants (Srivorakun et al., 2014). For G6PD, all detected variants were classified as pathogenic or likely pathogenic, consistent with prior functional and epidemiologic studies in Southeast Asia (Phompradit et al., 2011; Cappellini & Fiorelli, 2008; Luzzatto, Ally & Notaro, 2020). The accurate detection of heterozygous female carriers, with balanced allele fractions on IGV visualization, further supports the clinical applicability of long-read sequencing in X-linked disorders.

The current panel does not cover fetal hemoglobin (HbF) modifier loci, including the *Xmn*1-*HBG*2 polymorphism, *BCL11A*, *KLF*1, and *HBS1L-MYB* variants, which are key modulators of clinical severity in beta-thalassemia and sickle cell disease. While the present workflow focuses on pathogenic variant detection, future expanded panels incorporating these modifier loci would improve genotype–phenotype prediction for prenatal counseling and patient management.

One of the most compelling findings of our study is the frequent multilocus inheritance pattern. Several individuals harbored combinations of α-thalassemia, β-thalassemia, structural hemoglobin variants, and G6PD deficiency. Such multilayered genotypes may significantly influence hematologic phenotype, hemolytic risk, and transfusion requirements. Previous epidemiologic studies in Thailand have documented the coexistence of thalassemia and G6PD deficiency, particularly in malaria-endemic regions (Phompradit et al., 2011). However, routine diagnostics rarely evaluate both systems simultaneously. Our integrated long-read platform demonstrates a feasible strategy for comprehensive screening, which could be particularly valuable in prenatal settings or high-prevalence communities.

The ONT-based platform is best positioned as a first-line comprehensive test for complex clinical scenarios such as prenatal diagnosis and multi-locus workups; rather than a universal replacement for conventional methods. Gap-PCR and allele-specific PCR remain cost-effective for routine single-locus queries. Broader adoption will depend on local infrastructure, bioinformatics capacity, and instrument availability, which currently vary across diagnostic laboratories in Thailand and Southeast Asia. From a health-economic perspective, when samples are appropriately multiplexed, the per-sample cost of the ONT workflow—encompassing library preparation reagents, flow cell usage, and bioinformatics infrastructure—is competitive with the combined cost of gap-PCR, allele-specific PCR, and Sanger sequencing across multiple loci.

## Conclusion

Using the ONT long-read sequencing workflow, all samples were successfully genotyped across four clinically relevant genes in a single integrated platform. The method demonstrated 100% concordance with prior molecular diagnoses for both deletional and non-deletional variants, while simultaneously enabling comprehensive variant annotation and ACMG classification. Collectively, these results demonstrate that long-read nanopore sequencing provides a robust, high-resolution, and comprehensive approach for the simultaneous molecular characterization of thalassemia, hemoglobinopathies, and G6PD deficiency in a genetically heterogeneous population.

## Supplemental Information

10.7717/peerj.21605/supp-1 Supplemental Information 1 Raw data (variants).

10.7717/peerj.21605/supp-2 Supplemental Information 2 PCR primers.

10.7717/peerj.21605/supp-3 Supplemental Information 3 Raw data in each sample for Review.

## Additional Information and Declarations

### Data Availability

The following information was supplied regarding data availability:

The raw data of variants are available in the Supplemental File.

The sequencing data are accessible *via* NCBI BioProject: PRJNA1439314.
