# Supplementary material for: Simultaneous detection of thalassemia, hemoglobinopathies, and G6PD variants using long-read nanopore sequencing: genetic complexity and heterogeneity in Thailand
Source: PeerJ. 2026 Aug 11;14:e21605. doi: 10.7717/peerj.21605 (PMC13475388; doi:10.7717/peerj.21605)
Supplement: Supplementary file 1 [file peerj-14-21605-s002.docx]

**Supplement**

**Table 1 PCR primers**

| Primers | Sequence 5’-3’ |
| --- | --- |
| 3.7/20.5R | AAAGCACTCTAGGGTCCAGCG |
| A17F | GCTCCGCGCCAGCCAATGAG |
| α-2R | AGACCAGGAAGGGCCGGTG |
| GSF1 | CAATAGGGCCGGCTTGAC |
| GSR1 | TGCAACAATTAGTTGGAAAAGC |
| GSF2 | TGTCCCCAGCCACTTCTAAC |
| GSR3 | TGCAGGGTGACTGGCTCT |
| GSF4 | ACAGGGCGGGGAGCTAAG |
| GSR6 | GACAAGGAAGTGGGTCCTCA |
| L6A | GCAGAAACAGTTAGATGTCC |
| F13 | AATGCACTGACCTCCCACAT |
